# Spread of equine arteritis virus among Hucul horses with different EqCXCL16 genotypes and analysis of viral quasispecies from semen of selected stallions

**DOI:** 10.1038/s41598-020-59870-y

**Published:** 2020-02-19

**Authors:** Wojciech Socha, Pawel Sztromwasser, Magdalena Dunowska, Barbara Jaklinska, Jerzy Rola

**Affiliations:** 1 National Veterinary Research Institute, Al. Partyzantow 57, 24-100 Pulawy, Poland; 2 School of Veterinary Science, Massey University, Palmerston North, New Zealand; 3 Hucul Horse Stud Gladyszow, Regietow 28, 38-315 Uscie Gorlickie, Poland; 4 Medical University of Lodz, Al. Kosciuszki 4, 90-419 Lodz, Poland

**Keywords:** Viral evolution, Viral reservoirs

## Abstract

Equine arteritis virus (EAV) is maintained in the horse populations through persistently infected stallions. The aims of the study were to monitor the spread of EAV among Polish Hucul horses, to analyse the variability of circulating EAVs both between- and within-horses, and to identify allelic variants of the serving stallions EqCXCL16 gene that had been previously shown to strongly correlate with long-term EAV persistence in stallions. Serum samples (n = 221) from 62 horses including 46 mares and 16 stallions were collected on routine basis between December 2010 and May 2013 and tested for EAV antibodies. In addition, semen from 11 stallions was tested for EAV RNA. A full genomic sequence of EAV from selected breeding stallions was determined using next generation sequencing. The proportion of seropositive mares among the tested population increased from 7% to 92% during the study period, while the proportion of seropositive stallions remained similar (64 to 71%). The EAV genomes from different stallions were 94.7% to 99.6% identical to each other. A number (41 to 310) of single nucleotide variants were identified within EAV sequences from infected stallions. Four stallions possessed EqCXCL16S genotype correlated with development of long-term carrier status, three of which were persistent shedders and the shedder status of the remaining one was undetermined. None of the remaining 12 stallions with EqCXCL16R genotype was identified as a persistent shedder.

## Introduction

Equine viral arteritis (EVA) is one of the economically important diseases of horses and other equids^1^. The causative agent is equine arteritis virus (EAV), which has been recently reclassified within a species *Alphaarterivirus equid* in the genus *Alphaarterivirus* of the family *Arteriviridae* in the order *Nidovirales*^2^. Infection with EAV is often subclinical, but occasionally can lead to clinical disease of various severity after an incubation period of three to 14 days. The underlying cause is vascular injury, which leads to development of clinical signs that include pyrexia, depression, anorexia, dependent oedema, conjunctivitis, petechial haemorrhages on mucosal surfaces and urticaria. Pregnant mares may abort, and foals may develop severe interstitial pneumonia or pneumoenteric syndrome, depending on the age at the time of infection^1^.

Antibodies against EAV have been detected in equine sera worldwide, with only Japan, Iceland and New Zealand currently considered free from EAV infections^3^. The first reported outbreak of EVA in Poland occurred at one of the Thoroughbred studs in 1976–1977^4^. Since then, EAV specific antibodies have been detected in a high percentage of horses of different breeds throughout the country^5,6^. The virus has been implicated in economic losses, as can be illustrated by isolation of EAV from 23% of cases of abortion or neonatal death over a 34-year period in one Polish-based study^7^. The virus is transmitted between horses through either respiratory or venereal routes. Horizontal transmission occurs via infectious droplets of respiratory secretions from acutely infected horses or via direct contact between infectious and susceptible animals^1,8^. Although EAV is considered to be easily inactivated outside of the host, it can remain infective for as long as 75 days at 4 °C under laboratory conditions^9^, and lateral spread via fomites was speculated to occur under natural conditions by some authors^10^. Especially important for the epizootiology of EAV is venereal route of transmission through semen of a persistently infected stallion to susceptible mares. Between 10 and 70% of the infected stallions become persistent shedders for periods of time ranging from months to years (in some cases for the whole life of the animal)^1,8^. It has been recently shown that EqCXCL16 gene is correlated with the establishment of a long-term (>1 year) carrier status by EAV infected stallions. Those with at least one copy of the dominant allele (EqCXCL16Sa or EqCXCL16Sb) associated with *in-vitro* susceptibility of CD3 + T lymphocytes to EAV infection are more likely to become long-term shedders than those with two copies of the recessive allele (EqCXCL16R) linked to the resistant phenotype^11^.

The genome of EAV is a linear positive-sense single stranded RNA molecule that encodes 10 open reading frames (ORFs). The two most 5′ proximal ORFs (ORF1a and ORF1b) encode two overlapping polyproteins (1a and 1ab), which are further cleaved into 13 non-structural proteins that are essential for virus replication. The remaining eight ORFs (ORF2a, ORF2b, ORF3, ORF4, ORF5, ORF5a, ORF6 and ORF7) encode structural proteins of the virus^2^. In persistently infected stallions the virus undergoes mutations that accumulate over time, which may lead to the emergence of variants with increased virulence^12^.

The aims of the current study were: (1) to monitor the spread of EAV within a population of Hucul horses at one of the Polish national studs in the absence of targeted infection control measures; (2) to determine the variability of circulating EAVs, both within- and between EAV-infected Hucul horses; and (3) to determine the allelic variants of the serving stallions’ EqCXCL16 gene.

## Results

### EAV status of the sampled horses

Overall, 84/221 (38.0%) serum samples were positive for EAV antibody in the course of the study (Table 1). Out of 17 mares introduced to the stud in 2012, 14 tested negative for EAV antibody at both 05/2012 and 12/2012 samplings, while the remaining three had viral neutralisation test (VNT) titres that ranged between 8 (two mares) and 32 (one mare) at 05/2012, and remained the same six months later at the 12/2012 sampling. A sharp increase in the number of EAV seropositive mares ((χ^2^ = 48.7, p < 0.00001) was observed between 12/2012 and 05/2013 samplings. All 14 seronegative mares that were introduced to the stud in 2012 seroconverted to EAV by May 2013 with titres ranging from 32 to 128. A rising EAV titres were detected in the remaining three seropositive mares, with a four-fold or greater increase in the EAV titre observed for two of these mares. The proportion of EAV seropositive stallions remained similar within the same period ((χ^2^ = 0.45, P = 0.45).

**Table 1:** Rate of seropositivity to equine arterits virus (EAV) among Hucul horses at a Polish national stud between December 2010 and May 2013.

Sampling date	Sex	EAV seropositives			Average EAV titer (range)
		pos/sampled	%	95% CI^a^	
12/2010	All	6/21	28.6	8.8–48.4	257 (0–512)
	Mares	1/14	7.1	0–21.11	8 (0–8)
	Stallions	5/7	71.4	35.3–100	307 (0–512)
04/2011	All	6/20	30.0	9.4–50.6	343 (0–512)
	Mares	1/13	7.7	0–22.8	8 (0–8)
	Stallions	5/7	71.4	35.3–100	410 (0–512)
12/2011	All	7/27	25.9	9.1–42.8	212 (0–512)
	Mares	4/23	17.4	1.5–33.2	51 (0–128)
	Stallions	3/4	75.0	26.0–100	427 (0–512)
05/2012	All	11/51	21.6	10.2–33.0	190 (0–1024)
	Mares	4/37	10.8	0.7–21.0	28 (0–64)
	Stallions	7/14	50.0	22.8–77.2	283 (0–1024)
12/2012	All	11/51	21.6	10.2–33.0	172 (0–256)
	Mares	4/37	10.8	0.7–21.0	16 (0–32)
	Stallions	7/14	50.0	22.8–77.2	261 (0–256)
05/2013	All	43/51	84.3	74.3–94.4	113 (0–1024)
	Mares	34/37	91.9	83.0–100	60 (0–128)
	Stallions	9/14	64.3	38.2–90.3	313 (0–1024)
TOTAL	All	84/221	38.0	31.6–44.4	214 (0–1024)

^a^95% confidence interval.

Out of 37 semen samples from 11 stallions that were tested for EAV RNA, 21 were positive (Table 2). The positive samples were collected from six stallions, including three that tested positive more than once, and were hence classified as permanent shedders (hucPL2, hucPL3 and hucPL5).

**Table 2:** Equine arteritis virus (EAV) infection status for stallions at the Polish Hucul stud enrolled in the study based on detection of the virus in semen by RT-qPCR and detection of EAV antibodies by virus neutralisation tests (VNT).

Stallion	EAV RNA in semen (results/testing date)	EAV VNT (titre/sampling date)	EAV status^a^
hucPL1	Pos/May 2013	64/May 2013	S
hucPL2	Pos/Dec2010, Pos/Apr2011, Pos/Dec2011, Pos/May2012, Pos/Dec2012, Pos/May2013	512/Dec2010, 512/Apr2011, 256/Dec2011, 512/May2012, 256/Dec2012, 1024/May2013	PS
hucPL3	Pos/Dec2010, Pos/Apr2011, Pos/Dec2011, Pos/May2012, Pos/Dec2012, Neg/May2013	556/Dec2010, 256/Apr2011, 512/Dec2011, 512/May2012, 128/Dec2012, 512/May2013	PS
hucPL4	Pos/May2013	<4/May2012, 64/May2013	S
hucPL5	Pos/Dec2010, Pos/Apr2011, Pos/Dec2011, Pos/May2012, Neg/May2013	128/Dec2010, 256/Apr2011, 256/Dec2011, 1024/May2012, 256/May2013	PS
hucPL6	Neg/Apr2011, Neg/Dec2011, Neg/May2013	<4/Apr2011, <4/Dec2011, <4/May2013	N
hucPL7	Neg/Dec2010, Neg/Apr2011, Neg/Dec2011, Neg/May2013	<4/Dec2010, <4/Apr2011, <4/Dec2011, <4/May2013	N
hucPL8	Not tested	<4/May2012, <4/Dec2012, <4/May2013	N
hucPL9	Neg/May2013	64/Dec2012, 64/May2013	NS
hucPL10	Not tested	64/May2012, 32/Dec2012, 256/May2013	U
hucPL11	Pos/Dec2010, Neg/Apr2011, Neg/Dec2011, Neg/May2012, Neg/Dec2012, Neg/May2013	512/Dec2010, 512/Apr2011, 512/Dec2011, 512/May2012, 256/Dec2012, 512/May2013	NS
hucPL12	Not tested	<4/Dec2012, <4/May2013	N
hucPL13	Not tested	64/May2012, 64/Dec2012, 64/May2013	U
hucPL14	Not tested	<4/May2013	N
hucPL15	Neg/Dec2010	128/Dec2010, 256/Apr2011	NS
hucPL16	Neg/Dec2010	<4/Dec2010	N

^a^EAV status: S (shedder), PS (permanent shedder), N (not infected), NS (non-shedder, a serologically positive horse that is negative for the presence of the virus in semen), U (unknown).

### Genomic analysis of EAV

**Figure 1:**
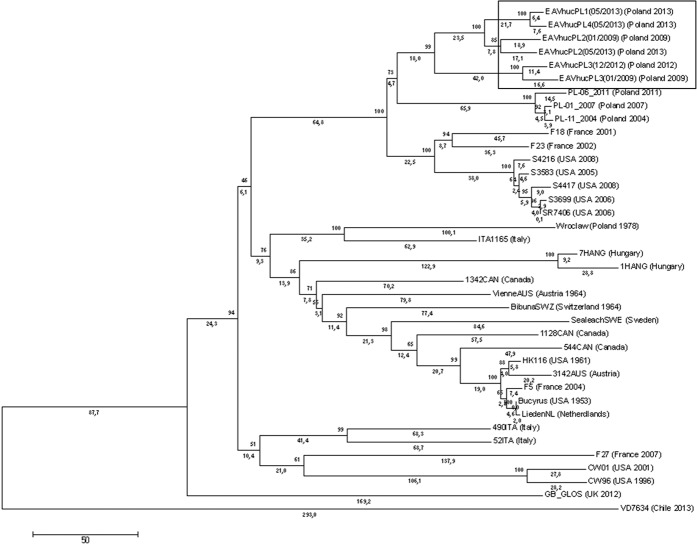
Neighbour-joining phylogenetic tree of sequences obtained in the study. The tree was constructed using a 2,895 nucleotide fragment of genomic RNA coding for structural genes of equine arteritis virus (EAV). Sequences acquired in this study are shown within a box. The following additional EAV sequences were included in the tree: PL-06_2011 (JX984459.1), PL-01_2007 (JX984455.1), PL-11_2004 (JX984449.1), F18 (EF492556.1), F23 (EF492561.1), S4216 (GQ903811.1), S3583 (GQ903809.1), S3699 (GQ903796.1), S4417 (GQ903798.1), SR7406 (GQ903866.1), ITA1165 (JN314874.1), 7HANG (JN314866.1), 1HANG (JN314862.1), 1342CAN (JN314858.1), SealeachSWE (JN314888.1), 1128CAN (JN314856.1), 544CAN (JN314854.1), 3142AUS (JN314851.1), LiedenNL (JN314875.1), 490ITA (JN314869.1), 52ITA (JN314867.1), VD7634 (MF598091.1), Wroclaw (JN314876.1), VienneAus (JN314852.1), Bibuna SWZ (JN314889.1), HK116 (EU586274.1), Bucyrus (NC_002532), F5 (EF492543.1), F27 (JN211316.1), CW96 (AY349167.1), CW01 (AY349168.1), GB_Glos (LC000003.1). Country and the date of isolation (when available) are included in the brackets next to each sequence. Numbers represent the average number of nucleotide substitutions between branches.

Full genome sequences of EAV were obtained from six semen samples from four infected stallions with an average coverage of 115.78X for EAVhucPL1(05/2013), 146.1X for EAVhucPL2(01/2009), 123.6X for EAVhucPL2(05/2013), 14.7X for EAVhucPL3(01/2009), 290.8X for EAVhucPL3(12/2012) and 26.3X for EAVhucPL4(05/2013). The nucleotide identity between genomic sequences of EAV from different horses varied from 94.7% to 99.6%. Based on the phylogenetic analysis, EAV sequences from the current study were closely related to other Polish EAV sequences from the last two decades (Fig. 1).

**Figure 2:**
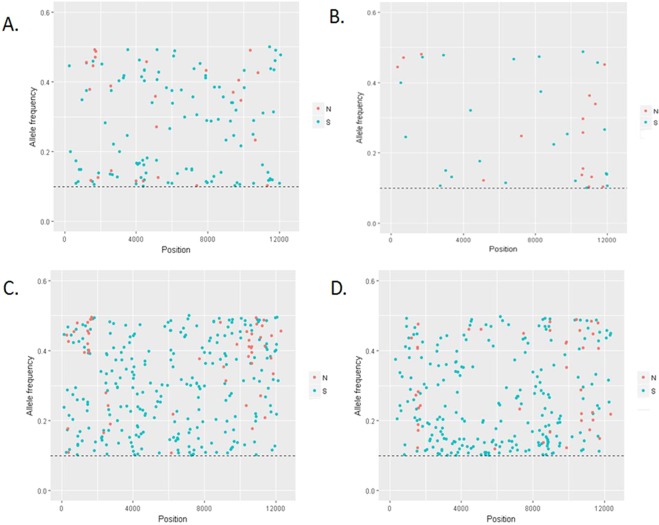
Frequency distribution of single nucleotide variants (SNVs) across an equine arteritis virus (EAV) genome. Synonymous (S) and non-synonymous (N) SNVs across the EAV sequences from EAVhucPL1(05/2013) (**A**), EAVhucPL2(01/2009) (**B**), EAVhucPL2 (05/2013) (**C**), and EAVhucPL3(12/2012) (**D**). Only SNVs with frequencies ≥10% and ≥5X coverage are shown.

Single nucleotide variants (SNVs) were analysed in order to determine the intra-host variability of EAV. The total coverage for sequences from EAVhucPL4(05/2013) and EAVhucPL3(01/2009) was considerably lower than the total coverage obtained for viral sequences from the remaining samples, and below a value of 100 recommended for variant detection studies with next generation sequencing (NGS)^13^. Hence, the NGS data for EAV sequences from hucPL4 was not included in the SNV analysis. Multiple variable sites were identified throughout the viral genomes from each of the remaining three stallions, with the highest number (n = 310) detected in EAVhucPL2(05/2013) and the lowest (n = 41) in EAVhucPL2(01/2009) (Table 3). Distribution of synonymous and non-synonymous SNVs is presented in Fig. 2. For all EAV sequences included in the analyses, the majority of non-synonymous SNVs were located in ORF1a fragment encoding non structural protein (nsp) 2 (Table 4) and in genes encoding viral surface glycoprotein (GP) 2, GP3, GP4 and GP5 (Table 5).

**Table 3:** Total number and distribution of synonymous and non-synonymous single nucleotide variants in EAV genomes from three infected stallions including two confirmed persistent shedders (hucPL2 and hucPL3, see Table 2).

gene	protein	position^a^	EAVhucPL1 (05/2013)			EAVhucPL2 (01/2009)			EAVhucPL2 (05/2013)			EAVhucPL3 (12/2012)		
			SNV	Syn	N-Syn	SNV	Syn	N-Syn	SNV	Syn	N-Syn	SNV	Syn	N-Syn
Leader		1–224	0			0			2			0		
ORF1ab		225–9751	110	90	20	19	14	5	237	210	27	217	198	19
ORF1a		225–5399	67	51	16	13	9	4	137	116	21	120	107	13
ORF1b		5405–9751	43	39	4	6	5	1	100	94	6	97	91	6
	nsp1	225–1004	9	9	0	4	2	2	24	19	5	12	12	0
	nsp2	1005–2717	24	14	10	3	2	1	54	40	14	48	37	11
	nsp3	2718–3416	5	5	0	3	3	0	15	14	1	17	17	0
	nsp4	3417–4028	5	4	1	0	0	0	16	16	0	17	17	0
	nsp5	4029–4514	13	12	1	1	1	0	15	15	0	11	10	1
	nsp6	4515–4580	2	1	1	0	0	0	3	3	0	2	2	0
	nsp7	4581–5255	9	6	3	2	1	1	7	7	0	8	7	1
	nsp8	5256–5405	0	0	0	0	0	0	3	3	0	5	5	0
	nsp9	5256–7333	16	16	0	3	2	1	43	42	1	38	36	2
	nsp10	7334–8734	15	13	2	2	2	0	29	28	1	33	31	1
	nsp11	8735–9391	6	6	0	1	1	0	16	13	3	25	22	3
	nsp12	9392–9748	6	4	2	0	0	0	12	12	0	6	6	0
ORF2a	E	9751–9954	3	3	0	1	1	1	3	3	0	5	4	1
ORF2b	GP2	9824–10507	6	5	1	1	1	0	14	10	4	7	3	4
ORF3	GP3	10306–10797	5	3	2	5	1	4	15	7	8	11	9	2
ORF4	GP4	10700–11158	3	2	1	4	1	3	15	12	3	11	8	3
ORF5a	5a	11112–11291	0	0	0	1	1	0	4	3	1	2	1	1
ORF5	GP5	11146–11913	12	11	1	6	2	4	22	14	8	18	11	7
ORF6	M	11901–12389	2	0	0	4	4	0	9	8	1	7	7	0
ORF7	N	12313–12645	2	0	0	0	0	0	2	1	1	2	1	1
**Total variant sites**			140			41			310			267		

^a^As compared to EAV Bucyrus strain (NC_002532).

Abbreviations: EVA – equine arteritis virus; SNVs – single nucleotide variants; Syn – synonymous; N-Syn – non-synonumous; ORF – open reading frame; nsp – non-structural protein; GP – glycoprotein; E – envelope protein; M – matrix protein; N – nucleocapsid protein.

**Table 4:** Non-synonymous single nucleotide variants in non-structural protein 2 (nsp2) of equine arteritis virus (EAV) from three infected stallions including two confirmed persistent shedders (hucPL2 and hucPL3, see Table 2).

nt^a^ pos	aa^b^ pos	EAVhucPL1 (05/2013)				EAVhucPL2 (01/2009)				EAVhucPL2 (05/2013)				EAVhucPL3 (12/2012)			
		Dom^c^	Alt^d^	Dom/Alt^e^	(%) Dom	Dom^c^	Alt^d^	Dom./Alt^e^	(%) Dom	Dom^c^	Alt^d^	Dom./Alt^e^	(%) Dom	Dom^c^	Alt^d^	Dom./Alt^e^	(%) Dom
968	323	K	R	25/21	54.4	R	—			R	—			R	K	119/92	56.4
1044	348	N	—			N	—			K	N	49/32	60.5	N	—		
1166	389	I	T	41/35	62.1	T	—			T	—			T	—		
1220	407	P	—			L	—			P	L	71/8	90.0	P	—		
1223	408	P	—			P	—			P	—			P	S	187/70	72.8
1226	409	E	—			G	—			G	E	43/36	54.4	G	—		
1236	412	E	D	60/8	88.8	K	—			K	—			K	—		
1244	415	V	—			E	—			E	V	42/39	61.9	E	—		
1282	428	A	—			A	—			A	T	58/38	60.4	A	—		
1292	431	S	—			S	—			S	I	58/39	59.8	N	—		
1298	433	A	—			A	—			A	V	55/45	55.0	A	—		
1310	437	T	—			T	—			T	—			T	I	151/105	59.0
1325	442	T	—			T	—			T	—			M	I/T	127/143	47.0
1354. 1355	452	S	P	41/33	55.4	P	—			P	—			P	Q/R/S	132/122	52.0
1369	457	T	—			T	—			T	—			A	S	191/59	76.4
1388	463	V	—			A	—			A	V	47/45	51.1	A	—		
1430	477	E	—			G	—			E	G	52/51	50.5	G	—		
1445	482	G	—			E	G	91/98	51.8	E	G	55/53	50.9	E	—		
1453	485	Y	H	23/22	51.0	H	—			H	—			H	—		
1463	488	R	Q	37/33	52.9	Q	—			Q	—			Q	—		
1490	497	A	—			A	—			A	—			A	V	203/66	75.5
1498	500	L	F	39/37	51.3	L	—			L	—			L	—		
1646	549	V	A	35/5	87.5	V	—			V	—			V	—		
2128	710	M	—			M	—			M	L	85/17	83.5	M	—		
2250	750	M	—			I	—			I	M	71/28	72.0	I	—		
2329	777	S	P	22/14	61.1	S	—			S	—			S	—		
2374. 2376	792	I	M	35/6	85.5	I	—			I	V	84/27	75.7	I	—		

^a^Nucleotide position in ORF1a gene (GenBank accession number NC_002532.2).

^b^Amino acid position in polyprotein 1ab (GenBank accession number NP_127506.1).

^c^Dominant amino acid variant.

^d^Alternative amino acid variant.

^e^Number of dominant and alternative variants present.

Percentage of dominant variants.

**Table 5:** Non-synonymous single nucleotide variants in genes encoding structural proteins of equine arteritis virus (EAV) from three infected stallions including two confirmed persistent shedders (hucPL2 and hucPL3, see Table 2).

ORF (Protein)	nt^a^ pos	aa^b^ pos	EAVhucPL1(05/2013)				EAVhucPL2(01/2009)				EAVhucPL2(05/2013)				EAVhucPL3(12/2012)			
			Dom^c^	Alt^d^	Dom/Alt^e^	Dom (%)^f^	Dom	Alt	Dom/Alt	Dom (%)	Dom	Alt	Dom/Alt	Dom (%)	Dom	Alt	Dom/Alt	Dom (%)
ORF2a (E)	145	49	A	—			A	—			A	—			T	A	187/26	87.8
ORF2b	8	3	R	—			R	—			R	L	68/49	58.1	R	—		
(GP2)	20	7	L	S	30/16	65.3	S	—			S	—			L	—		
	26	9	C	—			C	—			C	—			C	Y	129/94	57.8
	50	17	C	—			C	—			C	—			C	Y	129/95	57.6
	65	22	L	—			L	—			L	—			L	S	136/74	64.8
	608	203	Y	—			Y	—			Y	—			Y	C	209/202	51.1
	644	215	R	—			L	—			L	R	90/50	61.6	R	—		
	662	221	S	—			L	—			L	S	87/61	58.8	L	—		
	677	226	I	—			T	—			T	I	85/65	57.0	T	—		
ORF3	46	16	L	—			L	—			F	L	87/28	75.7	F	—		
(GP3)	79	27	R	—			R	—			R	S	59/50	54.5	S	—		
	85	29	Y	N	27/26	50.5	N	—			N	—			N	—		
	304	102	E	—			E	K	69/11	86.2	K	—			K	—		
	311	104	Y	—			F	—			Y	F	97/67	59.1	F	Y	210/178	54.2
	344. 345	115	S	R	59/18	76.4	N	S	76/14	84.4	S	N/K/R	96/67	58.9	E	—		
	346. 347	116	L	—			P	S/F/L	59/25	70.3	L	—			L	—		
	361. 362	121	Q	—			K	—			Q	K/T/P	91/73	55.2	K	—		
	373	125	K	—			K	—			K	E	139/30	82.2	K	E	292/118	71.3
ORF4	11	4	Y	—			Y	—			Y	—			Y	C	365/93	79.9
(GP4)	14	5	G	—			G	—			G	—			G	V	358/101	78.0
	109	37	I	F	31/26	57.5	F	—			F	—			I	F	220/155	59.2
	209	70	T	—			T	—			T	N	81/80	50.6	T	—		
	222	74	M	—			I	—			I	M	76/70	52.4	I	—		
	293	98	Q	—			R	Q	146/17	89.6	Q	—			Q	—		
	326	109	T	—			T	I	105/60	63.4	T	—			T	—		
	451	151	Y	—			Y	H	119/18	86.9	Y	H	86/32	72.9	Y	—		
ORF5a	140	47	V	—			V	—			A	V	65/58	52.8	A	—		
(ORF5a)	173	58	A	—			A	—			A	—			A	E	215/201	51.6
ORF5	5	2	L	—			L	S	119/18	86.9	L	S	86/32	72.9	L	—		
(GP5)	17	6	A	—			V	—			V	A	73/57	56.2	V	—		
	29	10	L	—			L	—			L	—			F	S	340/72	82.6
	182. 183	61	K	—			N	—			K	N	78/71	51.3	K	T	299/86	77.7
	200	67	T	M	88/11	89.8	T	—			T	—			T	—		
	217	73	V	—			V	L	91/72	66.0	V	—			I	V	207/168	55.2
	233	78	N	—			N	—			N	—			N	I	290/73	79.9
	244	82	N	—			N	—			N	D	84/59	58.7	N	—		
	251	84	H	—			H	—			Q	R	106/28	79.1	H	—		
	511	171	T	—			T	—			T	—			T	A	153/141	52.0
	520	174	V	—			V	—			V	—			I	V	175/122	59.3
	595	199	S	—			A	—			S	A	108/60	62.1	A	S	281/49	85.2
	622	208	V	—			I	—			V	I	93/74	55.7	I	—		
	626	209	Y	—			Y	F	146/17	89.6	Y	—			Y	—		
	704	235	V	—			V	A	97/75	55.8	V	A	100/50	66.7	A	—		
ORF6 (M)	401	134	N	—			N	—			N	S	62/52	54.4	N	—		
ORF7 (N)	32	11	A	—			A	—			A	—			A	V	179/50	78.2
	319	107	S	—			S	—			S	P	48/29	62.3				

^a^Nucleotide position in gene, ^b^Amino acid position in protein, ^c^dominant amino acid variant, ^d^alternative amino acid variant, ^e^number of dominant and alternative variants detected, ^p^ercentage of dominant variants.

**Figure 3:**
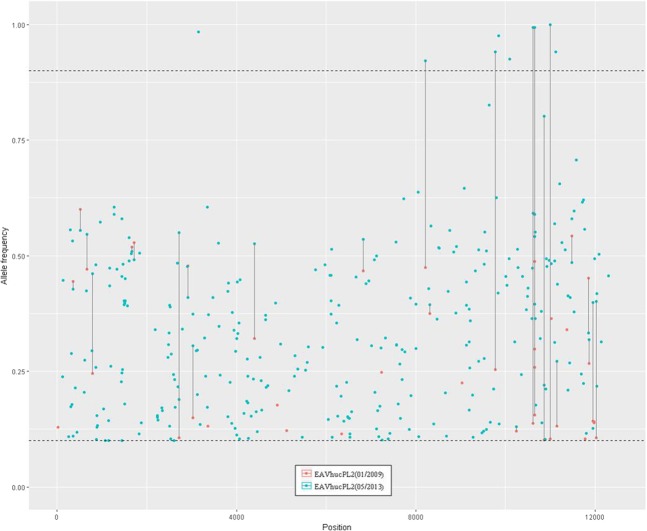
Frequency distribution of single nucleotide variants (SNVs) across an equine arteritis virus (EAV) genome during persistence in one host. Red dots - SNVs from EAVhucPL2(01/2009), blue dots - SNVs from EAVhucPL2(05/2013). Lines connect SNVs present both in 2009 and 2013, and show changes in their frequencies. Only SNVs with frequencies ≥10% and ≥5X coverage are shown.

Two samples collected four years apart were available from stallion hucPL2. Based on the analysis of these two samples, the number of SNVs increased from 41 to 310 between 2009 and 2013. Some (n = 12) low-frequency SNVs present in 2009 became predominant in 2013, while others (n = 13) disappeared (Fig. 3).

### EqCXCL16 genotypes of EAV-infected stallions

**Figure 4:**
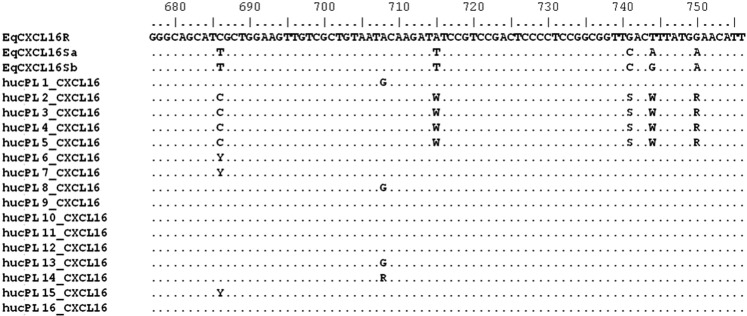
Allelic variants of EqCXCL16 gene detected among Hucul stallions enrolled in the study. Nucleotide positions 715, 741, 744 and 750 are determinants of CD3 + T lympocyte susceptibility to equine arteritis virus infection *in-vitro*^11^ that is correlated with the likelihood of development of EAV carrier satus. Stallions with two copies of recessive EqCXCL16R allele are more likely to clear the virus than stallions with at least one copy of the dominant EqCXCLSa or EqCXCLSb allele, which are more likely to develop persistent EAV infection. Sequences with ambigious bases on positions 715, 741, 744 and 750 were interpreted as representing a heterozygous (susceptible) genotype.

Among 16 stallions sampled in the study, 12 (75%) were homozygous for EqCXCL16R allele and hence had a genotype associated with resistance of CD3 + T lymphocytes to EAV infection and decreased likelihood of development of long-term carrier status, and four (hucPL2, hucPL3, hucPL4 and hucPL5) were heterozygotes (EqCXCL16R/EqCXCL16Sa) and hence were considered to have a susceptible phenotype correlated with an increased likelihood of establishment of long-term carrier status following EAV infection (Fig. 4).

## Discussion

Equine arteritis virus, similarly to other RNA viruses, is characterized by a high genetic variability. This results in generation of closely related genetic variants, known as quasispecies, within an individual host^14^. Until recently, quasispecies were identified by Sanger sequencing of cloned PCR products amplified from either viral isolates or directly from semen of infected horses^15^. In the current study NGS was used for sequence analysis of EAV. Six full consensus sequences of EAV from four different stallions were obtained, with the sequencing depth that allowed for identification of genetic variants appearing with as low as 10% frequency for four of them. Although multiple variable sites were found in each of the analysed viral sequences, their total number differed between EAVs from the three stallions. The lowest number (n = 41) was found in the 2009 EAV sequence from hucPL2, followed by the 2013 sample from hucPL1 (n = 140). Both numbers represented less than half of the SNVs identified in the EAV sequences obtained from the two remaining samples, including a sample from stallion hucPL2 collected in 2013. It has been previously shown that EAV remains genetically stable after the onset of infection and during early persistence, but its variability increases over time in response to pressures from the host’s immune system^16,17^. Our results support this conclusion, as hucPL3 and hucPL2 were persistently infected with on-going viral replication for at least six years prior to samplings in 2012/13, while hucPL1 was EAV negative until 2013. In addition, there was a clear increase in the intra-host diversity between EAV sequences from stallion hucPL2 collected four years apart (Table 3, Fig. 3).

The highest numbers of SNVs were identified in genes encoding nsp2, GP2, GP3, GP4 and GP5 of the virus. In general, this was consistent with the results of the previous studies using classical sequencing methods^12,15^. Similarly, in the most recent study that employed NGS, genes coding for GP3, GP5 and nsp2 showed the highest level of variability with the highest intra-host evolutionary rates recorded for ORF3 and ORF5, coding for GP3 and GP5^18^.

In agreement with the previous data^19^, non-synonymous SNVs in ORF5 were located predominantly in three variable regions (V) of GP5: V1 encompassing aminoacid (aa) positions 61–121, V2 (aa 141–178), and V3 (aa 202–222). The V1 has been previously shown to contain three out of four major neutralization sites (aa 49, 61, 67–90, and 99–106)^20^. Each of the analysed viruses showed non-synonymous SNVs in at least one of these four major neutralization sites within GP5^20^. Hence, our data support the previous assumption that variations in ORF5 is driven by the immune selection pressure^17^.

Non-synonymous SNVs were also found in genes coding for minor EAV glycoproteins (GP2, GP3 and GP4). It has been previously established that those proteins function in trimeric complexes. Hence, there are some structural constraints limiting their variability in regions responsible for binding^21^. This was consistent with our NGS results, as all non-synonymous SNVs in ORF2b (coding for GP2) were located in two previously described variable regions: V1 (aa 1–33) or V4 (aa 161–227)^22^. No SNVs were found at positions encoding cysteine (aa 48, 102, 137 and 195) in any of the viruses analysed, consistent with the importance of these residues for binding other minor glycoproteins^23^. All fixed amino acid changes within GP2 from a persistently infected stallion that was followed over a seven-year period in a previous study were also limited to the same two regions^22^.

Variability in GP3 was limited to two previously described highly variable sites (aa 3–41 and 98–121)^24^. It has been speculated that variants that had lost both glycosylation sites within the overlapping sequon (_28_NNTT_31_) through a change from asparagine to another amino acid at positions 28 and 29 may be selected during EAV persistence. This in turn could lead to the cleavage of the signal peptide in N-terminal end of GP3, although it is currently unknown what, if any, functional advantage would this create for the virus^25,26^. Our results do not support these speculations, as variants lacking one of the glycosylation sites (aa 29, Table 5) were found only in a recently infected stallion hucPL1, while asparagine was conserved at both positions in stallions hucPL2 and hucPL3 that have been persistently infected with EAV for years.

No variability was found in any of the glycoslation sites (aa 33, 55, 65 and 90) in predicted GP4 sequences. However, SNVs were found at nt positions 11 or 109 (corresponding to aa positions 4 and 37) in EAV sequences from two stallions. It has been proposed that these two SNVs may be involved in the attenuation of the vaccine strains of EAV^27^. Interestingly, substitution at aa position 37 of GP4 was found only in EAV from placenta of an infected pregnant mare, but not in EAV from any of the tested carrier stallions^28^. Therefore, the role of this SNV remains to be established, but it is unlikely that it is associated with carrier status of the host.

The only nsp where multiple SNVs were detected in the current study was nsp2. It has been previously shown that this protein induces humoral antibody responses in both naturally infected and vaccinated horses^29^. The majority of SNVs identified in EAVhucPL3(12/2012) and EAVhucPL2(05/2013) (two long-term carriers) were located in the part of ORF1a encoding a hypervariable region (aa 388–480)^12,30^. However, the pattern of SNVs in stallions that were recently infected with EAV at the time of sampling looked different. Most of SNVs in EAVhucPL1(05/2013) were located within the central region of nsp2, and only one SNV was present in nsp2 from EAVhucPL2(01/2009). Hence, it appears that SNVs accumulate within the hypervariable region of nsp2 during persistence, most likely due to selection driven by the immune response.

Next generations sequencing analysis performed in this study allowed for the determination of frequency and location of SNVs, and for the analysis of changes in the make-up of viral quasispecies within one persistently infected stallion over a period of time. Although this provided some insights into the changes in the dominant SNVs present at various time-points post-infection, a larger group of persistently infected stallions would need to be tested to confirm the observed tendencies and to determine whether or not some SNVs are linked to each other. It was not possible to reconstruct full-length sequences of individual viral haplotypes within the quasispecies population of EAV in each sample due to the use of short read sequencing. Application of novel NGS sequencing methods such as linked-read sequencing or long-read sequencing may allow to overcome this difficulty in the future^31^.

A number of environmental, viral and host factors may affect the outcome of EAV infection and establishment of persistent infection. These include factors such as breed of the horse, viral genotype or management^32,33^. Stallions in the current study were all of the same breed, managed under similar conditions, and were exposed to closely related variants of EAV. Hence, these factors were unlikely to be responsible for the observed differences in the duration of viral shedding by infected stallions. Results of two recent studies suggested that stallions with specific alleles of EqCXCL16 gene, which are linked to *in-vitro* susceptibility of CD3 + T lymphocytes to EAV infection, were at increased risk of becoming persistent shedders of the virus^11,32^. The majority (12/16) of stallions in the current study were homozygous for the resistant (EqCXCL16R) genotype. Unfortunately, data from reverse transcriptase quantitative polymerase chain reaction (RT-qPCR) and serology were available for only some of the stallions at each of the six sampling occasions, which is a limitation of the study. Nonetheless, 3/16 stallions (hucPL2, hucPL3 and hucPL5) tested positive for EAV RNA in the semen for a period of at least two years and as such, could be classified as persistent shedders. All three stallions had an EqCXCL16 genotype associated with *in vitro* susceptibility to EAV infection and development of long-term carrier status. One stallion (hucPL11) with resistant genotype was positive for EAV RNA at only one sampling occasion and negative at all subsequent samplings, and hence has presumably cleared the infection. For six stallions the shedder status could not be determined as none, or only one, semen sample was tested by RT-qPCR for the presence of the virus. This group included five stallions with a resistant genotype and one (hucPL4) with a susceptible genotype. The remaining six stallions with a resistant genotype did not show any evidence of EAV infection (were serologically negative) at the time(s) they were tested. Altogether, out of the four EAV positive stallions that could be confidently classified as shedders or non-shedders, only one stallion with the resistant EqCXCL16 genotype cleared the virus. The remaining three that became persistent shedders all had susceptible EqCXCL16 genotypes. This is in agreement with the results reported previously where 29/34 (85.3%) stallions with a resistant genotype cleared the virus following EAV infection, whereas 32/43 (74.4%) stallions with the susceptible genotype became persistent shedders^11^.

Serological analysis performed in this study was the extension of the previous study^6^ where EAV seropositivity among horses at the same Hucul stud was monitored between 2006 and 2008. In that study, the differences in EAV antibody status between various age groups were described, while in the current study we were interested in the changes in the serological status of horses over a period of time. For the first five samplings the percentage of EAV seropositive horses remained relatively low around 20–30%, compared with over 55% reported previously^6^. However, between 2012 and 2013 seroconversion was detected in the majority of the tested mares, which resulted in the increase of the EAV seropositive horses to over 90% at the last sampling. Based on the interview with the stud’s staff, at least four of the EAV infected stallions, including three persistently infected ones (hucPL2, hucPL3 and hucPL5), were used for breeding in 2012 and 2013. It is not unusual to use persistently infected stallions for reproduction^34^ providing that certain precautions are taken including ensuring that the stallions are bread only seropositive mares and such mares are separated for up to three weeks following cover^35^. Such precautions were not taken when a group of 17 EAV seronegative mares was introduced to the stud in 2012 and bred for the first time during 2012/13 breeding season. All these mares showed serological evidence of EAV infection between December 2012 and May 2013. It is likely that the initial spread of the virus was via venereal route, however subsequent spread probably also involved respiratory route and possibly fomites, as two previously seronegative stallions (hucPL1 and hucPL4) became EAV positive in 2013. The sharp increase in the proportion of EAV seropositive horses from 22% to 84% over a period of six months provides a good example of how quickly the virus can spread within any horse population if appropriate infection control precautions are not maintained.

In conclusion, the results of this study add to our understanding of EAV epidemiology and genomic variability. To our knowledge, this is the first study that involved horses of the primitive Hucul breed. We have shown that intra-host diversity of EAV sequences is concentrated at specific sites of the genome. The impact of these variations on the expression of disease and virus-host interaction needs to be elucidated in future research.

## Methods

### Horses and sampling

Hucul horses from a national horse stud located in the southern part of Poland were used for the study. Results of our previous study demonstrated the circulation of EAV within this herd^6^. Coagulated blood samples for serology were collected between December 2010 and May 2013 every six months from both mares and stallions. In total, 221 blood samples from 16 stallions and 46 mares were collected. These included samples from 17 mares that were introduced to the stud shortly before May 2012 sampling.

Semen was collected from seropositive stallions whenever possible and tested for the presence of viral RNA. Altogether, 35 semen samples from 11 stallions were collected. In addition, two archival semen samples from January 2009 from EAV positive horses hucPL2 and hucPL3 were included in the analyses. Eight stallions from the sampled group (hucPL1, hucPL2, hucPL3, hucPL5, hucPL8, hucPL10, hucPL11, and hucPL13) were used for breeding at least once during the period of the study.

As the sampling and testing was done as part of veterinary management of the stud, no additional approval of the Local Ethics Committee was required for these activities (Directive 2010/63/EU).

### Serology

Antibodies to EAV were detected using the VNT performed as described in the OIE Manual^36^. Briefly, serial two-fold dilutions of heat-inactivated (30 min at 56 °C) serum samples were added to each well of the 96-well microtitre plate in duplicate and mixed with 25 μL (100 to 300 tissue culture infectious dose 50% (TCID_50_)) of the Bucyrus strain of EAV diluted in Eagle’s minimum essential medium (MEM) (Sigma-Aldrich) containing guinea-pig complement at a final concentration of 10%. After incubation for 1 h at 37 °C, 50 μL (approximately 5 × 10^3^ cells) of rabbit kidney 13 (RK13) cells (ATCC CCL-37) were added to each well. The plates were sealed with parafilm and incubated for three to five days at 37 °C in a humidified atmosphere with 5% CO_2_. Appropriate virus-, cell- and serum controls were included with each test run. The neutralisation titres were expressed as reciprocals of the highest dilution of the serum that inhibited viral cytopathic effect (CPE). A titre of 4 or greater was considered positive.

### Viral RNA extraction and EAV-specific RT-qPCR

Total RNA was extracted directly from seminal plasma using QIAamp Viral RNA Mini Kit (Qiagen). Extractions were performed according to the manufacturer’s instruction with the exception that linear polyacrylamid (1 µL/sample) was used as carrier RNA. Reverse transcriptase qPCR was performed using Quantitect Viral Kit (Qiagen) and primers/probe complementary to a highly conserved region within the ORF7 gene of EAV as previously described^37^. Each reaction consisted of 1x QuantiTect Virus Master Mix, 1x QuantiTect Virus RT Mix, 0.8 µM of each primer, 75 nM of Taq Probe and 5 µL of template RNA in a total volume of 25 µL. The amplification conditions included the reverse transcriptase (RT) step (35 min at 48 °C), followed by the initial denaturation (10 min at 90 °C), and 40 cycles of denaturation (15 sec at 95 °C) and annealing/elongation (1 min at 60 °C). Samples were considered positive if quantification cycle (Cq) values were lower than 38. Extraction controls consisted of a Bucyrus strain of EAV (ATCC VR-796, positive control) and semen from an EAV seronegative stallion (negative control). Amplification controls consisted of RNA extracted from the Bucyrus strain (positive control) and water (non-template control). The RT-qPCR run was considered valid if all controls showed the expected results.

### Next-generation sequencing

Six samples from four EAV infected stallions were subjected to NGS. These were labelled as: EAVhucPL1(05/2013), EAVhucPL2(01/2009), EAVhucPL2(05/2013), EAVhucPL3(01/2009), EAVhucPL3(12/2012) and EAVhucPL4(05/2013) where letters were used for identification of the stallion and numbers in bracket represented month and year of sampling. Stallions hucPL2 and hucPL3 were positive for EAV RNA in their semen since at least 2006^6^ whereas stallions hucPL1 and hucPL4 tested EAV positive for the first time in 2013. Prior to sequencing, all samples were concentrated by ultracentrifugation. Briefly, 6 mL of seminal plasma was diluted five times in phosphate buffered saline pH 7.3 to 7.5 (PBS) and centrifuged at 3,000 × g for 10 min. Supernatant was then layered onto a 30% glycerol cushion (5 mL) in ultraclear thinwall polypropylene ultracentrifuge tube (Beckman). Following addition of PBS to the final volume of 35 mL, each sample was centrifuged for 2 h at 236,000 × g at 4 °C in a 70Ti fixed angle rotor (Beckman). Supernatant was removed and the pellet was resuspended in 0.5 mL of PBS. Viral RNA for NGS was extracted from this preparation using the same procedure as described above for RT-qPCR. Next generation sequencing was performed at Genomed SA using Illumina MiSeq Personal Sequencer.

### Analysis of NGS data

Quality of 2 × 250 base pairs (bp) Illumina reads was checked with FastQC (http://www.bioinformatics.babraham.ac.uk/projects/fastqc). Overlapping reads were merged using bbmerge v36.62^38^ and trimmed with Trimmomatic v0.36^39^ to remove adapters and low quality reads. Next, reads mapping to ribosomal RNA database SILVA v. 128^40^ were excluded (BWA MEM^41^ 0.7.12 with default parameters), and the remaining reads were assembled *de-novo* into contigs using SPAdes 3.9.0^42^. Contigs containing EAV genomic sequences were identified in each sample using an online version of the NCBI blastn program (v2.8.0)^43^, optimized for highly similar sequences (Megablast). The largest contig with an EAV reference sequence as the top hit was selected for futher analysis.

To identify SNVs within viral populations present in each sample, we used BWA MEM^41^ with default parameters to map trimmed reads to the reference EAV sequences. A consensus viral sequence obtained from the relevant *de-novo* assembly was used as the reference sequence for stallions PL1 and PL4, while EAV sequences from 2009 samples were used as reference sequences for stallions PL2 and PL3. Variants were called using LoFreq. 2.1.2 with default parameters^44^. Frequency distribution of SNVs across each viral genome was visualized using R 3.4.1^45^. We included only variant alleles evidenced by at least five reads (alternative allele depth; as defined in^46^) and present in at least 10% of the reads at a given *loci* (alternative allele frequency).

### Phylogeny

Neighbour-joining phylogenetic tree was constructed based on the alignment of 2,895 nt long fragments of the viral genome encoding structural proteins (positions 9751–12645 in Bucyrus reference strain - NC_002532) using MEGA5.2 software^47^. Viral sequences obtained in this study, and selected EAV sequences previously detected in Poland (n = 4) and other countries (n = 15) were included in the analysis.

### Analysis of EqCXCL16 gene variant of the stallions

Total DNA was extracted from seminal plasma or serum samples (when semen was not available) collected from each of the stallions using QIAamp DNA Mini Kit (Qiagen) according to the manufacturer’s instructions. PCR reactions were performed using JumpStart Taq DNA Polymerase (Sigma-Aldrich). Each reaction mix consisted of 0.4 µM of each primer (CXCL16-F and CXCL16-R^11^), 0.4 µM of dNTP mix, 1 µL of JumpStart Taq DNA Polymerase, and 2 µL of DNA in 1× reaction buffer in a total volume of 25 µL. Specific PCR products (280 bp) were sequenced using the same primers. EqCXCL16 sequences were assigned to a genotype based on the presence of specific nucleotides at positions 715, 741, 744 and 750 following alignment with the reference sequence (XM_001504756) as described previously^11^. Two susceptibility alleles (T, C, A, A and T, C, G, A) were designated EqCXCL16Sa and EqCXCL16Sb, respectively. The recessive allele associated with resistance to *in-vitro* EAV infection of and development of long-term carrier status the establishment of persistent EAV infection (EqCXCL16R) was characterized by A, G, T, and G at the specified positions. All analyses were performed using MEGA5.2 software^47^.

### Nucleotide sequence accession numbers

The Polish EAV sequences obtained in this study have been submitted to GenBank under accession numbers MN056351 - EAVhucPL1(05/2013), MN180159 - EAVhucPL2(01/2009) MN104892 - EAVhucPL2(05/2013), MN056352 - EAVhucPL3 (01/2009), MN204505 - EAVhucPL3(12/2012), MN056353 - EAVhucPL4(05/2013).

### Ethics approval and consent to participate

An informed approval was sought from stud managers before commencement of sampling. One of the roles of the National Veterinary Research Institute in Pulawy is monitoring of endemic diseases among Polish livestock. The sampling for the current study was performed within the scope defined by this role.

## Data Availability

The data that support the findings of this study are available from the corresponding author upon request.
